# Supplementary material for: From sequence to enzyme mechanism using multi-label machine learning
Source: BMC Bioinformatics. 2014 May 19;15:150. doi: 10.1186/1471-2105-15-150 (PMC4229970; doi:10.1186/1471-2105-15-150)
Supplement: Additional file 2 — Java code of ml2db. Additional file ml2db_code.tar.gz contains the Java source code to run the multi-label machine learning experiments and save the results to database. The code’s Javadoc is included. [file 1471-2105-15-150-S2.zip › additional file 2/ml2db/utils/doc/uk/ac/standrews/utils/main/collectionutils/ListUtils.html]

ListUtils


JavaScript is disabled on your browser.


- Overview
- Package
- Class
- Use
- Tree
- Deprecated
- Index
- Help

- Prev Class
- Next Class

- Frames
- No Frames

- All Classes

- Summary:
- Nested |
- Field |
- Constr |
- Method

- Detail:
- Field |
- Constr |
- Method


uk.ac.standrews.utils.main.collectionutils

## Class ListUtils

- java.lang.Object
- - uk.ac.standrews.utils.main.collectionutils.ListUtils

- ---

    

  ```
  public class ListUtils
  extends java.lang.Object
  ```

  Utilities to manipulate lists

  Version:
  :   4 Jan 2008

  Author:
  :   Luna De Ferrari luna.deferrari-at-ed.ac.uk

- - ### Constructor Summary

    Constructors

    Constructor and Description

    `ListUtils()`
  - ### Method Summary

    Methods

    | Modifier and Type | Method and Description |
    | --- | --- |
    | `static java.lang.String[]` | `addStringToArray(java.lang.String[] array, java.lang.String textToAdd)` Takes an array and adds a string to all elements |
    | `static java.util.ArrayList<java.lang.String>` | `addStringToArrayList(java.util.ArrayList list, java.lang.String textToAdd)` Takes an arrayList and adds a string to all elements |
    | `static java.util.Set<java.lang.String>` | `cleanSet(java.util.Set<java.lang.String> set)` Cleans a set from null and empty elements |
    | `static java.util.LinkedHashSet` | `getCommonElements(java.util.ArrayList list1, java.util.ArrayList list2)` Gets the elements present in both lists |
    | `static int` | `getLongestStringLength(java.util.ArrayList<java.lang.String> array)` Get the length of the longest string in an array |
    | `static int` | `getShortestStringLength(java.util.ArrayList<java.lang.String> array)` Get the length of the shortest string in an array |
    | `static boolean` | `isNullOrEmpty(java.lang.String string)` tells if a string is null or empty |
    | `static boolean` | `isNullOrEmpty(java.util.Vector<java.lang.String> list)` tells if a list is null or empty |
    | `static void` | `printList(java.util.List list)` Prints an array as a list of 'index' + 'array element' (with a header containing the size) |
    | `static void` | `printListsOfLists(java.util.List<java.util.List> list)` Prints lists of lists |
    | `static java.util.TreeSet<java.lang.String>` | `setFromList(java.util.ArrayList<java.lang.String> list)` create a set (elements appear only once) from a list |
    | `static java.util.Vector<java.lang.String>` | `vectorFromList(java.util.ArrayList<java.lang.String> list)` |

    - ### Methods inherited from class java.lang.Object

      `equals, getClass, hashCode, notify, notifyAll, toString, wait, wait, wait`

- - ### Constructor Detail


    - #### ListUtils

      ```
      public ListUtils()
      ```
  - ### Method Detail


    - #### addStringToArray

      ```
      public static java.lang.String[] addStringToArray(java.lang.String[] array,
                                        java.lang.String textToAdd)
      ```

      Takes an array and adds a string to all elements


    - #### addStringToArrayList

      ```
      public static java.util.ArrayList<java.lang.String> addStringToArrayList(java.util.ArrayList list,
                                                               java.lang.String textToAdd)
      ```

      Takes an arrayList and adds a string to all elements


    - #### cleanSet

      ```
      public static java.util.Set<java.lang.String> cleanSet(java.util.Set<java.lang.String> set)
      ```

      Cleans a set from null and empty elements

      Parameters:
      :   `set` -

      Returns:
      :   a set without the null element or the empty string


    - #### getCommonElements

      ```
      public static java.util.LinkedHashSet getCommonElements(java.util.ArrayList list1,
                                              java.util.ArrayList list2)
      ```

      Gets the elements present in both lists

      Parameters:
      :   `list1` - a list
      :   `list2` - another list

      Returns:
      :   the set containing the common elements (each element only once)


    - #### getLongestStringLength

      ```
      public static int getLongestStringLength(java.util.ArrayList<java.lang.String> array)
      ```

      Get the length of the longest string in an array

      Parameters:
      :   `array` - an array of strings

      Returns:
      :   the length of the longest string


    - #### getShortestStringLength

      ```
      public static int getShortestStringLength(java.util.ArrayList<java.lang.String> array)
      ```

      Get the length of the shortest string in an array

      Parameters:
      :   `array` - an array of strings

      Returns:
      :   the length of the shortest string


    - #### isNullOrEmpty

      ```
      public static boolean isNullOrEmpty(java.lang.String string)
      ```

      tells if a string is null or empty

      Parameters:
      :   `string` - a string

      Returns:
      :   true if the string is null or empty (no characters), false
          otherwise


    - #### isNullOrEmpty

      ```
      public static boolean isNullOrEmpty(java.util.Vector<java.lang.String> list)
      ```

      tells if a list is null or empty

      Parameters:
      :   `list` -

      Returns:
      :   true if the list is null or empty, false otherwise


    - #### printList

      ```
      public static void printList(java.util.List list)
      ```

      Prints an array as a list of 'index' + 'array element' (with a header
      containing the size)

      Parameters:
      :   `list` - the List to print


    - #### printListsOfLists

      ```
      public static void printListsOfLists(java.util.List<java.util.List> list)
      ```

      Prints lists of lists

      Parameters:
      :   `list` -


    - #### setFromList

      ```
      public static java.util.TreeSet<java.lang.String> setFromList(java.util.ArrayList<java.lang.String> list)
      ```

      create a set (elements appear only once) from a list


    - #### vectorFromList

      ```
      public static java.util.Vector<java.lang.String> vectorFromList(java.util.ArrayList<java.lang.String> list)
      ```


- Overview
- Package
- Class
- Use
- Tree
- Deprecated
- Index
- Help

- Prev Class
- Next Class

- Frames
- No Frames

- All Classes

- Summary:
- Nested |
- Field |
- Constr |
- Method

- Detail:
- Field |
- Constr |
- Method
